# Maternal smoking around birth is associated with an increased risk of offspring constipation: Evidence from a Mendelian randomization study

**DOI:** 10.18332/tid/203866

**Published:** 2025-05-08

**Authors:** Yong Shen, Siqi Xie, Yu Lin, Yifan Fang, Bing Zhang, Jinna Zhang

**Affiliations:** 1 Fujian Children’s Hospital (Fujian Branch of Shanghai Children’s Medical Center), College of Clinical Medicine for Obstetrics & Gynecology and Pediatrics, Fujian Medical University, Fuzhou, China; 2 Fujian Maternity and Child Health Hospital College of Clinical Medicine for Obstetrics & Gynecology and Pediatrics, Fujian Medical University, Fuzhou, China

**Keywords:** maternal smoking around birth, constipation, childhood constipation, tobacco smoke expose, Mendelian randomization

## Abstract

**INTRODUCTION:**

This study aimed to investigate the association between maternal smoking around birth and the incidence of offspring constipation.

**METHODS:**

Genome-wide association study (GWAS) data for maternal smoking around birth and offspring constipation were obtained from the Mendelian randomization (MR) Base platform. Single nucleotide polymorphisms (SNPs) significantly associated with maternal smoking around birth were utilized as instrumental variables in two-sample MR analyses to explore the relationship between maternal smoking and offspring constipation. The analytical methods employed included the inverse-variance weighted (IVW) method, weighted median estimator, and MR-Egger regression.

**RESULTS:**

Twenty SNPs significantly associated with maternal smoking around birth (p<5×10^-8^; linkage disequilibrium r^2^<0.001) were identified. Across the different methods, a consistent positive association was observed between maternal smoking around birth and an increased risk of constipation in offspring (IVW: OR=4.35; 95% CI: 1.81–10.45; weighted median estimator: OR=4.23; 95% CI: 1.22–14.75; MR-Egger: OR=0.92; 95% CI: 0.01–122.07), suggesting that higher frequency of maternal smoking is associated with an elevated risk of constipation in offspring. However, we did not detect any potential effect of genetic liability to constipation risk on maternal smoking.

**CONCLUSIONS:**

This study provides evidence suggesting that increased maternal smoking around the time of birth may be linked to a higher risk of constipation in offspring.

## INTRODUCTION

Constipation is a common gastrointestinal symptom in children, with an incidence rate of approximately 1–30%^1^. It often begins in infancy or early childhood, with about one-third of affected children experiencing symptoms that persist into adolescence, placing a significant medical and psychological burden on the child and its family^2^. Childhood constipation is primarily categorized into organic and functional types, with the exact causes still unclear. It is now widely believed that changes in the gut microbiota can affect bowel motility and lead to constipation. Thus, modifying the diversity of gut microbiota may alleviate constipation, while disruptions in the enteric nervous system (ENS) also play a crucial role in its development^3^. The relationship between the gut microbiota and ENS in constipated children is quite complex, with increasing evidence indicating a sophisticated communication mechanism between them, and the gut immune system playing a significant role in this interaction^4^. The ENS and gut immune system form a complex enteric neuroimmune network, and changes in the gut microbiota can modulate this network, affecting the development of the host’s ENS and leading to alterations in gut function, which are important factors in the onset of constipation.

Smoking is a major global public health threat, causing over 8 million deaths worldwide each year. Despite increased awareness of the harmful effects of smoking and ongoing efforts to control tobacco use, 22.3% of the global population still smokes regularly^5^, including a portion of pregnant women. Smoking by mothers during the perinatal period has numerous adverse effects on fetal and child health. Despite various smoking cessation measures, around 11% of women continue to smoke during the perinatal period, exposing their unborn and newborn children to smoke^6^. Exposure to cigarettes affects the intestinal microbiota of children, leading to dysbiosis^7,8^ and causing abnormal bowel function and constipation. Changes in the gut microbiota may impact the ENS in children, contributing to the development of constipation^9^. Additionally, interactions with the host’s immune responses via the gut-neuroimmune network can influence the ENS, potentially leading to constipation in children^4^.

Previous studies examining the association between maternal smoking and offspring constipation have yielded inconclusive results, often confounded by unmeasured variables and susceptible to reverse association. While traditional randomized controlled trials (RCTs) offer methodological rigor, their ethical constraints limit feasibility in this context. Recently, Mendelian randomization (MR) has emerged as a robust approach for inference, leveraging genetic variation as an instrumental variable to address the limitations inherent in conventional epidemiological studies. In this study, we employed data from genome-wide association studies (GWAS) to perform a two-sample MR analysis, aiming to elucidate the relationship between maternal smoking around birth (ukb-b-17685) and the occurrence of constipation (ebi-a-GCST90018829). To enhance the robustness of our findings, estimates from two independent cohorts were combined, providing a more reliable assessment of the potential effect.

## METHODS

### Data source

**Figure 1:**
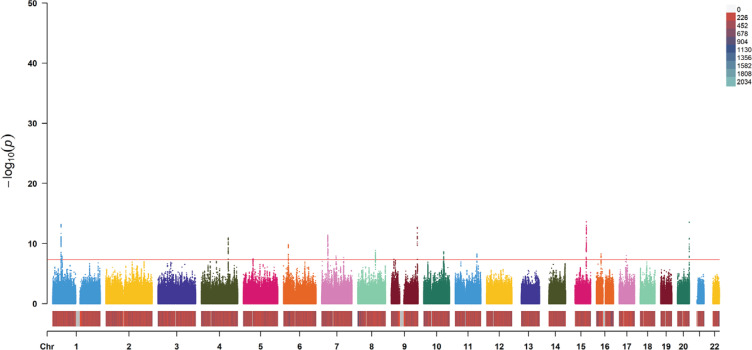
Manhattan plot of the included single nucleotide polymorphisms about maternal smoking around birth, data from IEU OpenGWAS in 2018 (SNPs=9851867; red line as threshold; p=5×10^-8^)

The design of the two-sample Mendelian randomization (MR) study is illustrated in Supplementary file Figure 1. We extracted summary data of genetic associations with maternal smoking around birth from the MRC Integrative Epidemiology Unit (MRC-IEU) consortium, published in 2018 and accessible via the UK Biobank^10^. This consortium included a total of 397732 participants and analyzed 9851867 single nucleotide polymorphisms (SNPs) (Figure 1).

The constipation dataset was sourced from the European Bioinformatics Institute (EBI)^11^, comprising 15902 cases and 395721 controls, with a total of 24176599 SNPs. Detailed information regarding the studies and datasets is presented in Table 1.

**Table 1:** Details of studies and datasets used in the study, data from 2018 and 2021 (N=397732 for maternal smoking around birth; N=411623 for constipation)

*Exposure/outcomes*	*Web source*	*Sample size*	*SNP size*	*Reference*	*Consortium*	*Year*	*Population studied*
**Maternal smoking around birth**	1787: Output from GWAS pipeline using *Phesant* derived variables from UKBiobank (ukb-b-17685)	397732	9851867	[10]	MRC-IEU	2018	Europe
**Constipation**	EMBL-EBI (ebi-a-GCST90018829)	411623	24176599	[11]	NA	2021	Europe

NA: not available.

### Selection of instrumental variables

Instrumental variables (IVs) for this study were selected based on the following criteria: 1) a significant genome-wide association (p<5×10^-8^) with the exposure and a minor allele frequency (MAF) >0.01 in the outcome; and 2) low linkage disequilibrium (LD) with r^2^<0.001 within a 10000 kb distance. SNPs associated with potential confounders or outcomes were identified using PhenoScanner^12^. Ultimately, 20 SNPs were included in the analysis (rs12405972, rs35566160, rs36072649, rs4865667, rs2183947, rs10226228, rs62477310, rs7002049, rs1323341, rs75596189, rs7899608, rs2428019, rs576982, rs12923476, rs6011779). The variance for each SNP was calculated using the formula:

R^2^=2 × β^2^ × EAF × (1-EAF) / [2 × β^2^ × EAF × (1-EAF) + 2 × SE^2^ × N × EAF × (1-EAF)]

where EAF is the effect allele frequency (EAF). The F-statistic was calculated from:

F=[(N-k-1)/k]×R^2^/(1-R^2^)

where N is the GWAS sample size, k is the number of IVs, and R^2^ is the proportion of exposure variance explained by the IVs. An F-statistic <10 suggests weak IVs, which may introduce bias into the results^13^. SNPs significantly associated with constipation are shown in Supplementary file Table 1.

### Main analysis method

Following the acquisition of data associated with maternal smoking around birth or constipation from GWAS studies via the MR-Base platform^14^, Mendelian randomization (MR) analysis was conducted using the TwoSampleMR package (version 0.5.8) within the R statistical software (version 4.3.2). Three distinct statistical approaches were employed: the inverse-variance weighted (IVW) method, weighted median estimator, and MR-Egger regression, to elucidate the relationship between maternal smoking around birth and constipation^15-18^. The IVW method involves meta-analyzing the Wald ratios of the included SNPs to assess the associations, assuming all included SNPs are valid^15,16^. In contrast, MR-Egger regression is based on the assumption of instrument strength independent of direct effect (InSIDE), and is robust to the inclusion of invalid SNPs^15^. The slope of the MR-Egger regression indicates the effect of maternal smoking around birth on constipation when the intercept term is zero or statistically insignificant^15,18^. The weighted median estimator requires at least 50% of the variables to be valid and reports results as odds ratios (ORs) with 95% confidence intervals (CIs). Statistical significance was determined for p<0.05. Reporting followed the STROBE-MR guidelines^19^.

### Sensitivity analysis

To assess the sensitivity of the results, we applied the leave-one-out method, wherein each SNP was systematically excluded one at a time, and the effects of the remaining SNPs were recalculated using the IVW method^20^. This rigorous approach enabled a comprehensive exploration of the influence of individual SNPs on the overall inference.

## RESULTS

### Detail information of the included SNPs

Table 2 gives detailed information on each SNP, including the effect allele (EA) and its frequency (EAF) in the exposure, as well as the estimates of their associations with maternal smoking around birth and constipation, including β values, standard errors (SE), and corresponding p values.

**Table 2:** Association analysis for maternal smoking around birth-increasing GWAS risk alleles with the offspring constipation, IEU OpenGWAS 2018 and 2021 (N=809355)

*CHR*	*Position*	*SNPs*	*EA*	*EAF*	*Maternal smoking around birth*			*Constipation*		
					*β*	*SE*	*p*	*β*	*SE*	*p*
1	44097438	rs12405972	T	0.348302	-0.00806	0.001075	6.39E-14	0.0026	0.0119	0.8251
2	164928199	rs35566160	G	0.27477	0.006373	0.001165	4.49E-08	-0.0171	0.0129	0.1852
4	140939110	rs36072649	A	0.380845	-0.00717	0.001057	1.13E-11	-0.0324	0.0117	0.0057
5	50748173	rs4865667	T	0.387775	-0.00581	0.001053	3.42E-08	-0.0051	0.0119	0.6710
6	26159356	rs2183947	A	0.224954	-0.00784	0.001225	1.54E-10	-0.0191	0.0136	0.1592
7	32315613	rs10226228	G	0.370229	0.007375	0.001063	3.97E-12	0.0152	0.012	0.2036
7	114951541	rs62477310	C	0.486743	-0.00578	0.00103	2.00E-08	-0.0226	0.0114	0.0473
8	93114414	rs7002049	C	0.7847	0.007562	0.00125	1.44E-09	0.011	0.0142	0.4402
9	14453010	rs1323341	G	0.781645	-0.00683	0.001243	3.89E-08	-0.0202	0.0136	0.1356
9	136468701	rs75596189	T	0.109986	0.012053	0.001642	2.15E-13	0.0127	0.0203	0.5312
10	104727304	rs7899608	T	0.141291	0.008782	0.001471	2.34E-09	0.0138	0.0163	0.3954
11	113678423	rs2428019	A	0.239291	0.007023	0.001202	5.08E-09	0.0096	0.0134	0.4718
15	78870803	rs576982	T	0.227864	-0.00933	0.001222	2.28E-14	-0.022	0.0132	0.0942
16	24798079	rs12923476	A	0.256949	-0.00686	0.001173	4.82E-09	-0.0059	0.0133	0.6544
20	61984317	rs6011779	T	0.808714	-0.00994	0.001304	2.50E-14	0.0026	0.0142	0.8532

EA: effect allele. EAF: effect allele frequency. SE: standard error. SNPs: single-nucleotide polymorphisms.

### The effect of maternal smoking around birth on offspring constipation

**Figure 2:**
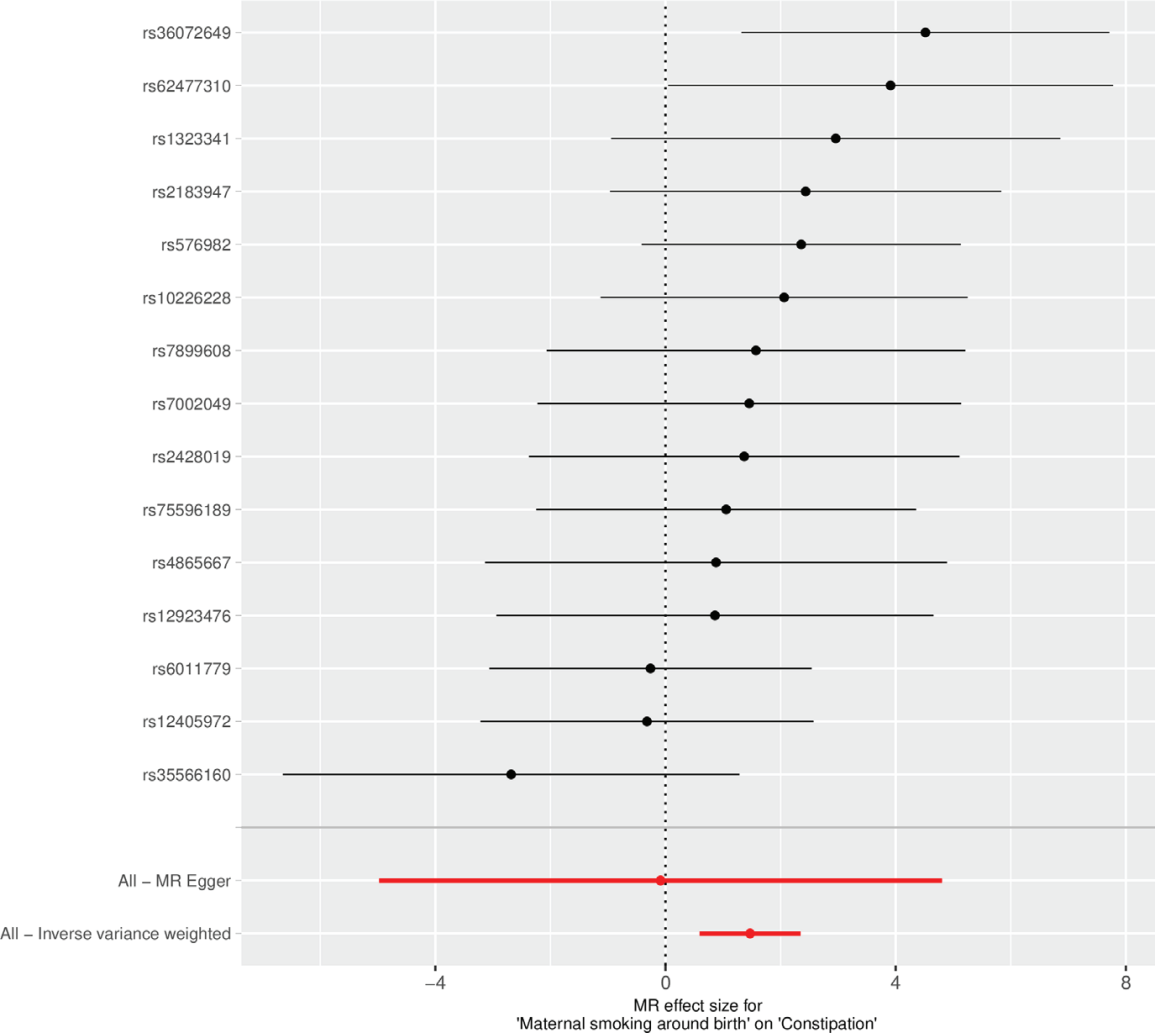
Forest plot of single nucleotide polymorphisms (SNPs) associated with maternal smoking around birth and the risk of constipation. Black points represent the log odds ratio (OR) for offspring constipation per standard deviation (SD) increase in maternal smoking around birth, data from 2018 and 2021 (N=397732 for maternal smoking around birth; N=411623 for constipation)

**Figure 3:**
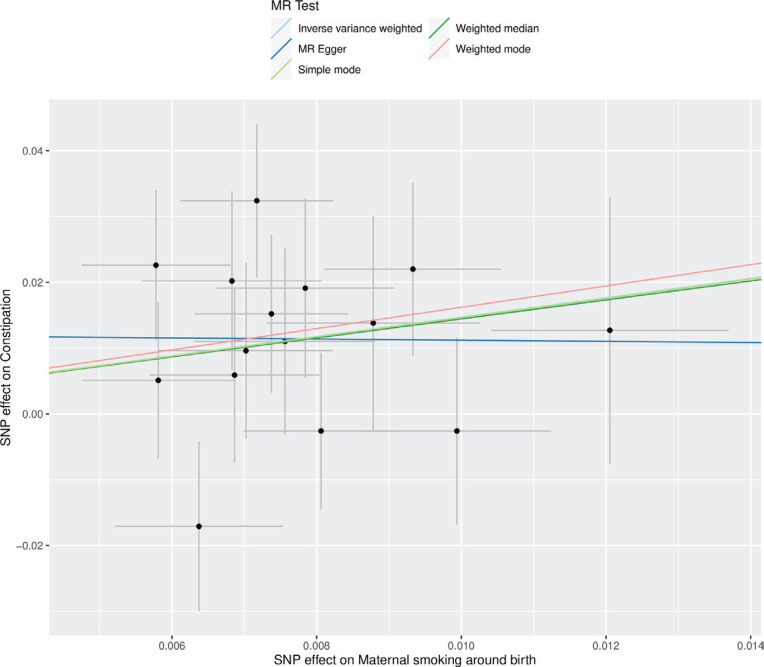
Scatter plot of the SNPs associated with maternal smoking around birth and the risk of constipation. The plot shows the effect sizes of SNP associations with maternal smoking (x-axis, in standard deviation units) and SNP associations with constipation (y-axis, log odds ratio), along with 95% confidence intervals. The regression slopes of the lines represent estimates derived from the three primary Mendelian randomization (MR) methods: the IVW method, weighted median estimator, and MR-Egger regression, IEU OpenGWAS 2018 and 2021 (N=809355)

The findings, presented in Table 3, demonstrate a positive association between maternal smoking around birth and an increased genetic predisposition to offspring constipation (OR=4.35; 95% CI: 1.81–10.45). Consistent results were obtained using the weighted median estimator^21^ (OR=4.23; 95% CI: 1.22–14.75) and the MR-Egger method (OR=0.92; 95% CI: 0.01–122.07). These results are further illustrated in the forest plot (Figure 2) and scatter plot (Figure 3)^22^.

**Table 3:** Associations between genetically determined MR analysis of exposures with outcomes, IEU OpenGWAS 2018 and 2021 (N=809355)

*Exposure*	*Outcome*	*Forward MR*	*OR (95% CI)*	*p*
Maternal smoking around birth	Constipation	MR Egger	0.92 (0.01–122.07)	0.972
		Weighted median	4.23 (1.22–14.75)	0.023
		IVW	4.35 (1.81–10.45)	0.001
		Simple mode	4.32 (0.49–37.93)	0.207
		Weighted mode	5.05 (0.59–42.81)	0.159
***Exposure***	***Outcome***	***Reverse MR***	***OR (95% CI)***	***p***
Constipation	Maternal smoking around birth	MR Egger	1.01 (0.99–1.03)	0.266
		Weighted median	1.01 (1.00–1.02)	0.143
		IVW	1.00 (0.99–1.01)	0.653
		Simple mode	0.99 (0.96–1.01)	0.333
		Weighted mode	1.01 (1.00–1.02)	0.127

IVW: inverse variance weighted. SE: standard error. Using R 4.3.2 software and TwoSampleMR R packages (version 0.5.8): OR < generated odds ratios (MR results).

### Sensitivity analysis

**Figure 4:**
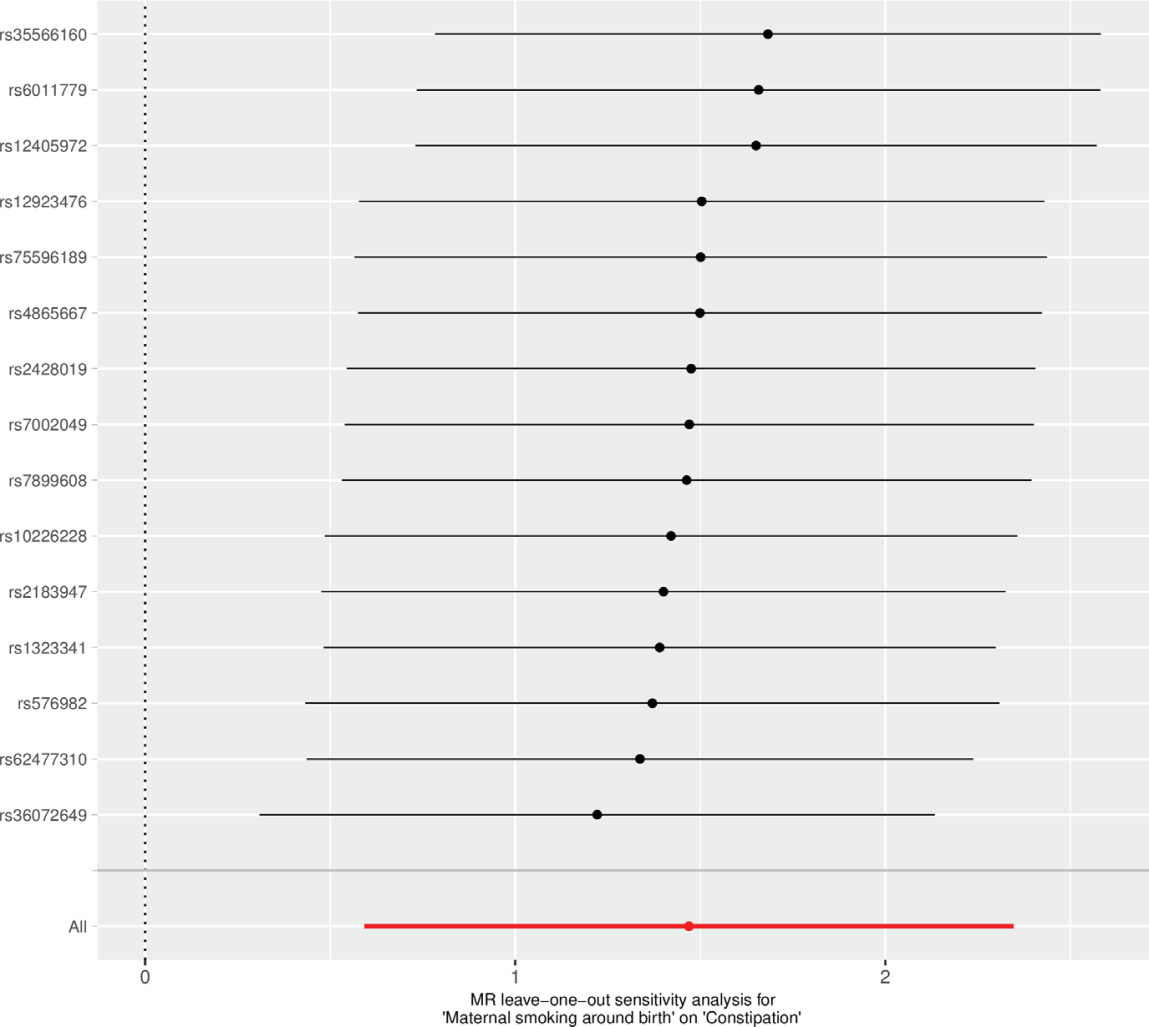
Leave-one-out of SNPs associated with maternal smoking around birth and their risk of constipation. Each black point represents result of the IVW MR method applied to estimate the effect of maternal smoking around birth on constipation excluding particular SNP, IEU OpenGWAS 2018 and 2021 (N=809355)

Genetic pleiotropy did not significantly impact the results, as evidenced by the MR-Egger regression intercept (0.012062, SE=0.019, p=0.417) (Table 4), the MR-Presso method was employed to detect outliers. If outliers were identified, they were removed, and the analysis was repeated. Additionally, neither the MR-Egger method nor the IVW method, as assessed by Cochran’s Q test, showed significant heterogeneity among the instrumental variables (IVs). The leave-one-out analysis further confirmed that no single SNP exerted a disproportionate influence on the inference (Figure 4).

**Table 4:** Heterogeneity and pleiotropy analyses, IEU OpenGWAS 2018 and 2021 (N=809355)

*MR analyses*	*Exposure*	*Outcome*	*Heterogeneity test*				*Pleiotropy test*
			IVW Q	p	MR-Egger Q	p	MR-Egger p
**The forward**	ukb-b-17685	ebi-a-GCST90018829	13.818	0.463	13.403	0.537	0.417
**The reverse**	ebi-a-GCST90018829	ukb-b-17685	24.324	0.060	22.369	0.071	0.287

IVW: inverse variance weighted. MR: Mendelian randomization.

### The reverse MR

We were unable to conduct a reverse MR analysis to explore a potential relationship between offspring constipation and maternal smoking around birth due to an insufficient number of IVs related to constipation as the exposure, which met the significance threshold of p<5×10^-8^. We derived 16 independent genetic instrumental variables (IVs) using a less stringent threshold of p<5×10^-6^ (linkage disequilibrium r^2^<0.001 within a 10000 kb distance). Neither MR-Egger nor inverse-variance weighted (IVW) methods revealed significant pleiotropy among these 16 independent IVs, as indicated by Cochran’s Q statistics (Table 4). The IVW analysis results suggested a lack of detectable effect of offspring constipation on maternal smoking around birth (Table 3). Furthermore, the leave-one-out sensitivity analysis revealed heterogeneity for each SNP when compared with other SNPs (Supplementary file Figure 2).

## DISCUSSION

Maternal smoking during the perinatal period exposes the fetus and newborn to tobacco smoke, disrupting gut microbiota homeostasis^21^. Research by Qu et al.^23^ revealed that smoke exposure significantly increased the abundance of *Intestinimonas* in the gut microbiota of mice. Similarly, Nolan et al.^24^ found that smokers had a higher abundance of *Catenibacterium* in their gut microbiota, with a positive correlation to smoking intensity. Lin et al.^25^ observed a reduction in the abundance of the *Ruminococcaceae* family among smokers in a cohort of 116 healthy Chinese men. Additionally, Wang et al.^26^ reported that smoking significantly decreased *Lactococcus* levels, with a negative correlation to the age at which smoking exposure began. These findings collectively suggest that maternal smoking profoundly impacts gut microbiota composition and functionality in children exposed to tobacco smoke.

The gut microbiota plays a crucial role in maintaining intestinal function and is a key component of the gut microenvironment^27^. Gut microbiota colonization begins during the fetal period^28^ and stabilizes within the first few weeks after birth. The gut microbiota influences the enteric nervous system (ENS) through various mechanisms, potentially affecting gut function. Anitha et al.^29^ demonstrated that germ-free mice exhibited reduced numbers of intestinal neurons and impaired gut motility compared to normal mice, effects that were partially reversible through fecal microbiota transplantation. Obata et al.^30^ further showed that the gut microbiota modulates the development and maturation of the ENS, thereby influencing gut function. Disruptions in gut microbiota can impact the gut’s neuroimmune network, potentially leading to constipation. Niu et al.^9^ found significant differences in gut microbiota between children with constipation and asymptomatic children, including reduced microbial diversity and increased relative abundance of *Ruminococcaceae* and *Bacteroidaceae*. These microbiota alterations may affect ENS function, contributing to constipation. Given that the ENS in children has greater developmental potential than in adults, dysbiosis during this critical period can disrupt ENS maturation, impair gut motility, and increase the risk of constipation^31^. Studies have shown that polycyclic aromatic hydrocarbons (PAHs) in tobacco can induce oxidative stress in the placenta^32^, which may trigger a chronic inflammatory response in the fetal intestine. Such inflammation may damage the intestinal mucosal barrier, leading to immature intestinal development or intestinal motility disorders.

This study represents a novel investigation into the interaction between maternal smoking around the time of birth and the incidence of constipation in offspring. Utilizing forward Mendelian randomization (MR) analysis, we assessed the impact of maternal smoking on offspring constipation, finding no significant heterogeneity or pleiotropy among the instrumental variables (IVs). The use of PhenoScanner confirmed that none of the selected SNPs exhibited significant pleiotropic effects, thereby validating the IVs used in the analysis. Furthermore, the independence of exposure and outcome datasets in this two-sample MR approach enhances the robustness and comprehensiveness of our findings. We identified twenty SNPs significantly associated with maternal smoking around birth as IVs. Employing the inverse-variance weighted (IVW) method, weighted median estimator, and MR-Egger regression with data from genome-wide association studies (GWAS) on constipation, our forward MR analysis across two independent GWAS datasets suggests a relationship between increased maternal smoking frequency and a heightened risk of constipation in offspring. Conversely, the analysis indicates no significant effect of offspring constipation on maternal smoking behavior around birth. These findings underscore the importance of further research to determine whether early interventions targeting maternal smoking could potentially reduce the risk of constipation in offspring.

The Mendelian randomization (MR) method employed in this study effectively controls for confounding factors and mitigates reverse association, drawing on data from published GWAS and meta-analyses. These sources provide a robust foundation, with substantial sample sizes and diverse genetic variations.

### Limitations

Several limitations in this study must be acknowledged^22^. Firstly, the validation of genetic polymorphisms remains challenging, and despite the application of the MR-Egger method, gene-environment interactions not assessed and potential misclassification cannot be entirely ruled out. Secondly, our analysis is based on a GWAS dataset of maternal smoking around birth derived from a European population, which may introduce bias due to population and ethnic stratification. Extending these findings to other populations will require further investigation. Thirdly, the two-sample MR approach may be susceptible to overidentification, which may result in potential violations of MR assumptions, leading to an overestimation of the association between SNPs and exposure. The wide confidence interval suggests inevitable imprecision due to the small sample size. Additionally, the UKBiobank and EMBL-EBI dataset used do not specify the severity of constipation in offspring, limiting our ability to explore the relationship between maternal smoking and various constipation subtypes. Finally, the diagnostic criteria for constipation are relatively subjective, which could introduce variability into the findings. Unobserved pleiotropy cannot be addressed.

## CONCLUSIONS

This study employed Mendelian randomization (MR) analysis to evaluate the potential impact of maternal smoking around birth on the incidence of offspring constipation. These results underscore the importance of identifying and protecting populations prone to maternal smoking, particularly children exposed to tobacco smoke, potentially informing novel strategies for the prevention and alleviation of offspring constipation. However, due to the possibility of residual confounding and other factors which may impact the genetic assessment, further research is necessary.

## Supplementary Material



## Data Availability

The data supporting this research are available from the authors on reasonable request.
