# Supplementary material for: Maternal smoking around birth is associated with an increased risk of offspring constipation: Evidence from a Mendelian randomization study
Source: Tob Induc Dis. 2025 May 8;23:10.18332/tid/203866. doi: 10.18332/tid/203866 (PMC12060150; doi:10.18332/tid/203866)
Supplement: Supplementary file 1 [file TID-23-58-s1.pdf]

Supplementary Table Part 1.

	A	B	C	D	E	F	G	H
1	beta.exposure	se.exposure	samplesize.exposure	SNP	effect_allele.exposure	other_allele.exposure	eaf.exposure	exposure
2	-0.0592	0.0128	397732	rs2354178	A	G	0.75465	exposure
3	0.0757	0.0162	397732	rs56029819	C	T	0.150442	exposure
4	-0.1576	0.0323	397732	rs74287578	C	A	0.0967724	exposure
5	0.2218	0.0456	397732	rs75271724	G	T	0.0167112	exposure
6	-0.2466	0.0512	397732	rs186529831	T	C	0.0197501	exposure
7	0.0546	0.0119	397732	rs10223052	G	A	0.566463	exposure
8	-0.0706	0.014	397732	rs1376767	C	A	0.756497	exposure
9	-0.0631	0.0135	397732	rs616812	C	T	0.725106	exposure
10	0.0565	0.0118	397732	rs4083752	A	G	0.455926	exposure
11	-0.0526	0.0114	397732	rs10139484	T	G	0.545353	exposure
12	-0.0811	0.0176	397732	rs79086497	A	G	0.120788	exposure
13	0.0546	0.0114	397732	rs11648475	G	A	0.48264	exposure
14	0.0558	0.0115	397732	rs12934865	T	C	0.460637	exposure
15	0.6488	0.1333	397732	rs139180659	C	T	0.007975	exposure
16	-0.0585	0.0121	397732	rs5757641	G	A	0.363219	exposure
17	0.1641	0.0286	397732	rs55694472	T	C	0.0433177	exposure
18	-0.0718	0.0148	397732	rs59674167	T	C	0.19228	exposure

Supplementary Table Part 2.

	I	J	K	L	M	N	O
1	mr_keep.exposure	pval.exposure	pval_origin.exposure	id.exposure	data_source.exposure	R2	F
2	TRUE	3.75E-06	inferred	JWDK6v	textfile	0.001297792	534.8925648
3	TRUE	2.97E-06	inferred	JWDK6v	textfile	0.001464819	603.8346621
4	TRUE	1.06E-06	inferred	JWDK6v	textfile	0.004342013	1795.057957
5	TRUE	1.15E-06	inferred	JWDK6v	textfile	0.001616746	666.5642911
6	TRUE	1.46E-06	inferred	JWDK6v	textfile	0.002354628	971.5017266
7	TRUE	4.47E-06	inferred	JWDK6v	textfile	0.001464242	603.5967588
8	TRUE	4.59E-07	inferred	JWDK6v	textfile	0.001836331	757.2629151
9	TRUE	2.95E-06	inferred	JWDK6v	textfile	0.001587287	654.3994035
10	TRUE	1.68E-06	inferred	JWDK6v	textfile	0.001583723	652.9277004
11	TRUE	3.95E-06	inferred	JWDK6v	textfile	0.001371998	565.5191345
12	TRUE	4.07E-06	inferred	JWDK6v	textfile	0.001396977	575.8292851
13	TRUE	1.67E-06	inferred	JWDK6v	textfile	0.001488783	613.7281121
14	TRUE	1.22E-06	inferred	JWDK6v	textfile	0.001547171	637.834983
15	TRUE	1.13E-06	inferred	JWDK6v	textfile	0.006660472	2759.972738
16	TRUE	1.33E-06	inferred	JWDK6v	textfile	0.001583071	652.6584572
17	TRUE	9.59E-09	inferred	JWDK6v	textfile	0.002231929	920.7637372
18	TRUE	1.23E-06	inferred	JWDK6v	textfile	0.001601304	660.1875843

STROBE-MR checklist of recommended items to address in reports of Mendelian randomization studies[1 2]

Item No.	Section		Checklist item	Page No.	Relevant text from manuscript
1	**TITLE and ABSTRACT**		Indicate Mendelian randomization (MR) as the study's design in the title and/or the abstract if that is a main purpose of the study	1	TITLE and ABSTRACT
	**INTRODUCTION**				
2	**Background**		Explain the scientific background and rationale for the reported study. What is the exposure? Is a potential causal relationship between exposure and outcome plausible? Justify why MR is a helpful method to address the study question	3	Line 27-30
3	**Objectives**		State specific objectives clearly, including pre-specified causal hypotheses (if any). State that MR is a method that, under specific assumptions, intends to estimate causal effects	4	Line 4-9
	**METHODS**				
4	**Study design and data sources**		Present key elements of the study design early in the article. Consider including a table listing sources of data for all phases of the study. For each data source contributing to the analysis, describe the following:		
		a)	Setting: Describe the study design and the underlying population, if possible. Describe the setting, locations, and relevant dates, including periods of recruitment, exposure, follow-up, and data collection, when available.	4	Line13-24
		b)	Participants: Give the eligibility criteria, and the sources and methods of selection of participants. Report the sample size, and whether any power or sample size calculations were carried out prior to the main analysis	4	Line13-24
		c)	Describe measurement, quality control and selection of genetic variants	4	Line13-24
		d)	For each exposure, outcome, and other relevant variables, describe methods of assessment and diagnostic criteria for diseases	4	Table1
		e)	Provide details of ethics committee approval and participant informed consent, if relevant		N/A

5	**Assumptions**	Explicitly state the three core IV assumptions for the main analysis (relevance, independence and exclusion restriction) as well assumptions for any additional or sensitivity analysis	4	Line 27-30
6	**Statistical methods: main analysis**	Describe statistical methods and statistics used		
	a)	Describe how quantitative variables were handled in the analyses (i.e., scale, units, model)	5	Line 2-4
	b)	Describe how genetic variants were handled in the analyses and, if applicable, how their weights were selected	5	Line 5-10
	c)	Describe the MR estimator (e.g. two-stage least squares, Wald ratio) and related statistics. Detail the included covariates and, in case of two-sample MR, whether the same covariate set was used for adjustment in the two samples	5	Line 12-26
	d)	Explain how missing data were addressed	5	Line 2
	e)	If applicable, indicate how multiple testing was addressed	5	Line 5-7
7	**Assessment of assumptions**	Describe any methods or prior knowledge used to assess the assumptions or justify their validity	5	Line 5-10
8	**Sensitivity analyses and additional analyses**	Describe any sensitivity analyses or additional analyses performed (e.g. comparison of effect estimates from different approaches, independent replication, bias analytic techniques, validation of instruments, simulations)	5-6	28-19,1-3
9	**Software and pre-registration**			
	a)	Name statistical software and package(s), including version and settings used	5	Line 15
	b)	State whether the study protocol and details were pre-registered (as well as when and where)		
	**RESULTS**			
10	**Descriptive data**			
	a)	Report the numbers of individuals at each stage of included studies and reasons for exclusion. Consider use of a flow diagram	6	Line 7-13, Table 2
	b)	Report summary statistics for phenotypic exposure(s), outcome(s), and other relevant variables (e.g. means, SDs, proportions)	6	Table 2

		c)	If the data sources include meta-analyses of previous studies, provide the assessments of heterogeneity across these studies		N/A
		d)	For two-sample MR:<br>i. Provide justification of the similarity of the genetic variant-exposure associations between the exposure and outcome samples<br>ii. Provide information on the number of individuals who overlap between the exposure and outcome studies	6	Line 17-22, Table 3
11	**Main results**				
		a)	Report the associations between genetic variant and exposure, and between genetic variant and outcome, preferably on an interpretable scale	6	Table 3
		b)	Report MR estimates of the relationship between exposure and outcome, and the measures of uncertainty from the MR analysis, on an interpretable scale, such as odds ratio or relative risk per SD difference	6	Table 3
		c)	If relevant, consider translating estimates of relative risk into absolute risk for a meaningful time period		N/A
		d)	Consider plots to visualize results (e.g. forest plot, scatterplot of associations between genetic variants and outcome versus between genetic variants and exposure)	6-7	Figure 2,3
12	**Assessment of assumptions**				
		a)	Report the assessment of the validity of the assumptions	7	Line 9-14
		b)	Report any additional statistics (e.g., assessments of heterogeneity across genetic variants, such as I^2, Q statistic or E-value)	7	Table 4
13	**Sensitivity analyses and additional analyses**				
		a)	Report any sensitivity analyses to assess the robustness of the main results to violations of the assumptions	7	Line 9-14
		b)	Report results from other sensitivity analyses or additional analyses	7	Line 9-14
		c)	Report any assessment of direction of causal relationship (e.g., bidirectional MR)	7	Line 23-27
		d)	When relevant, report and compare with estimates from non-MR analyses		N/A
		e)	Consider additional plots to visualize results (e.g., leave-one-out analyses)	7	Figure 4

	**DISCUSSION**				
14	**Key results**		Summarize key results with reference to study objectives	8-9	Line 28-30, 1-16
15	**Limitations**		Discuss limitations of the study, taking into account the validity of the IV assumptions, other sources of potential bias, and imprecision. Discuss both direction and magnitude of any potential bias and any efforts to address them	9	Line 20-30
16	**Interpretation**				
		a)	Meaning: Give a cautious overall interpretation of results in the context of their limitations and in comparison with other studies	9	Line 17-20
		b)	Mechanism: Discuss underlying biological mechanisms that could drive a potential causal relationship between the investigated exposure and the outcome, and whether the gene-environment equivalence assumption is reasonable. Use causal language carefully, clarifying that IV estimates may provide causal effects only under certain assumptions	8	Line 11-27
		c)	Clinical relevance: Discuss whether the results have clinical or public policy relevance, and to what extent they inform effect sizes of possible interventions	10	Line 6-9
17	**Generalizability**		Discuss the generalizability of the study results (a) to other populations, (b) across other exposure periods/timings, and (c) across other levels of exposure	9	Line 20-30
	**OTHER INFORMATION**				
18	**Funding**		Describe sources of funding and the role of funders in the present study and, if applicable, sources of funding for the databases and original study or studies on which the present study is based		In the submission system
19	**Data and data sharing**		Provide the data used to perform all analyses or report where and how the data can be accessed, and reference these sources in the article. Provide the statistical code needed to reproduce the results in the article, or report whether the code is publicly accessible and if so, where		In the submission system
20	**Conflicts of Interest**		All authors should declare all potential conflicts of interest		In the submission system

This checklist is copyrighted by the Equator Network under the Creative Commons Attribution 3.0 Unported (CC BY 3.0) license.

1. Skrivankova VW, Richmond RC, Woolf BAR, Yarmolinsky J, Davies NM, Swanson SA, et al. Strengthening the Reporting of Observational Studies in Epidemiology using Mendelian Randomization (STROBE-MR) Statement. JAMA. 2021;under review.

2. Skrivankova VW, Richmond RC, Woolf BAR, Davies NM, Swanson SA, VanderWeele TJ, et al. Strengthening the Reporting of Observational Studies in Epidemiology using Mendelian Randomisation (STROBE-MR): Explanation and Elaboration. BMJ. 2021;375:n2233.

Supplementary Figure 1.

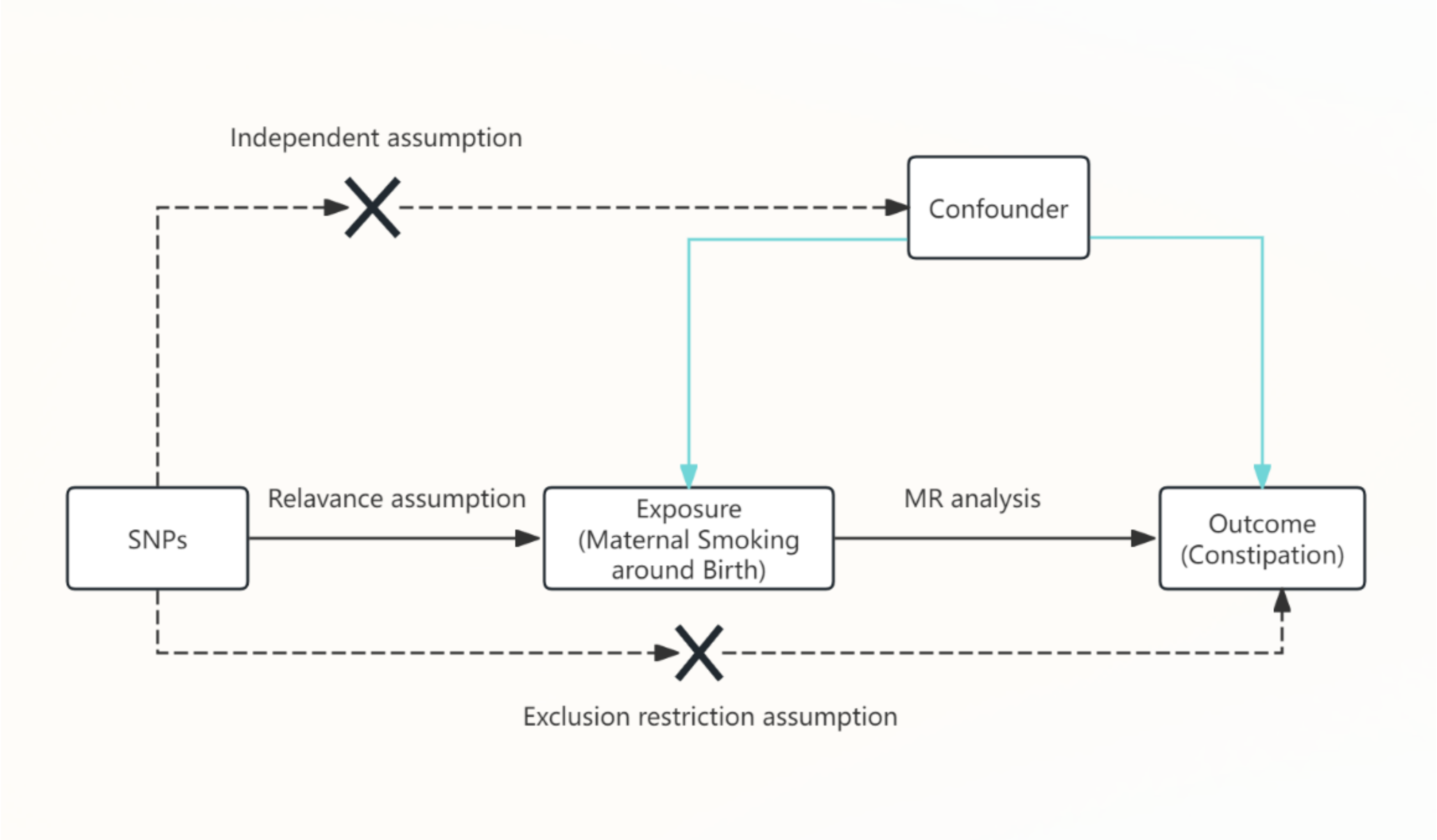

© 2025 Shen Y. et al.
